# Urticarial Vasculitis Induced by Adalimumab Biosimilar in an Elderly Female With Hidradenitis Suppurativa: A Case Report

**DOI:** 10.7759/cureus.57722

**Published:** 2024-04-06

**Authors:** Miasser Alarnaouti, Sultan Alfaer, Mawaddah Tallab, Asmaa A Khojah, Talah Magadmi, Abdulaziz Kadasa, Maha A Binsaqr

**Affiliations:** 1 Department of Dermatology, King Fahad General Hospital, Jeddah, SAU; 2 Family Medicine, Suliman Fakeeh Hospital, Jeddah, SAU; 3 College of Medicine, King Abdulaziz University, Jeddah, SAU; 4 General Practice, Ministry of Health, Qassim, SAU

**Keywords:** skin allergy, drug rash, urticarial vasculitis, adalimumab-adaz, hidradenitis suppurativa

## Abstract

Urticarial vasculitis (UV) is a type of small-vessel vasculitis, which is rarely associated with anti-tumor necrosis factor (TNF)-alpha medication. We describe a 72-year-old woman with multiple comorbidities on several medications, including an adalimumab biosimilar for Hurley stage II recalcitrant hidradenitis suppurativa (HS), who presented with new-onset severe angioedema and a rash with urticarial wheals that covered most of her body surface area. The diagnosis of drug-induced UV is supported by both the history of adalimumab biosimilar use and the histopathology result. The patient responded successfully to a course of doxycycline administered for three months, which was preceded by corticosteroid dosages, both orally and intravenously, to reduce inflammation. The given case highlights the correlation between a distinct dermatologic autoimmune manifestation and TNF-targeted therapy, demonstrating the importance for dermatologists to be aware of the potential side effects of adalimumab biosimilars in order to manage them effectively.

## Introduction

A rare small-vessel vasculitis known as urticarial vasculitis (UV) is characterized by recurrent episodes of persistent wheal-like lesions that resemble those seen in chronic spontaneous urticaria (CSU) but tend to last longer than 24 hours. The lesions also exhibit histopathologic characteristics of leukocytoclastic vasculitis and heal, leaving a residual ecchymotic post-inflammatory hyperpigmentation [1]. UV can just affect the skin, but it can also affect other organs, including the kidneys, lungs, gastrointestinal tract, and eyes, and can present with other constitutional symptoms like fever and arthralgia. UV can be divided into two main groups depending on the serum complement level, normo-complementemic UV (NUV), which is more common, and hypo-complementemic UV (HUV), which is the more severe form. Many UV cases are those of unknown reasons but can be related to other factors, such as drugs or underlying diseases, including infections, malignancy, and autoimmune or auto-inflammatory disease [2]. UV is more common in females, especially between the age of 40 and 60. The absence of significant randomized or controlled trials examining the effectiveness of current treatments for urticarial vasculitis can make it challenging to treat the condition. The U.S. Food and Drug Administration has not yet approved any medications for use in treating this illness [3].

## Case presentation

This was a case of a 72-year-old Saudi female, a known case of diabetes mellitus type 2 on Metformin, with osteoporosis, degenerative scoliosis of the spine, bronchial asthma, on a regular inhaler, who had a seizure attack on carbamazepine started six weeks before her presentation to the hospital, hidradenitis suppurativa (HS), recalcitrant type, and Hurely stage II on adalimumab 40 mg once weekly. For the last year, she was shifted to adalimumab-adaz, which is a tumor necrosis factor (TNF)-alpha-blocker that is biosimilar to adalimumab, 40 mg weekly for one month. The last dose of adalimumab-adaz received was on February 16, 2023.

On February 27, 2023, she presented to the emergency department (ED) with severe angioedema and rash accompanied by fever, chills, cough, shortness of breath, sore throat, and dysphagia. The patient was admitted to the medical ward for 10 days as a case of severe angioedema with a rash secondary to a drug reaction. The rash was not aggravated by sun exposure, there was no oozing of fluid, blood, or pus discharge, and she denied skin changes that define bullae or purpura. Systematic evaluation was significant for oliguria, and dysuria, reduced hearing, and excessive watery discharge bilaterally from both ears. In addition to fatigue, loss of appetite, and weight loss of 5 kilograms in one week.

**Figure 1:**
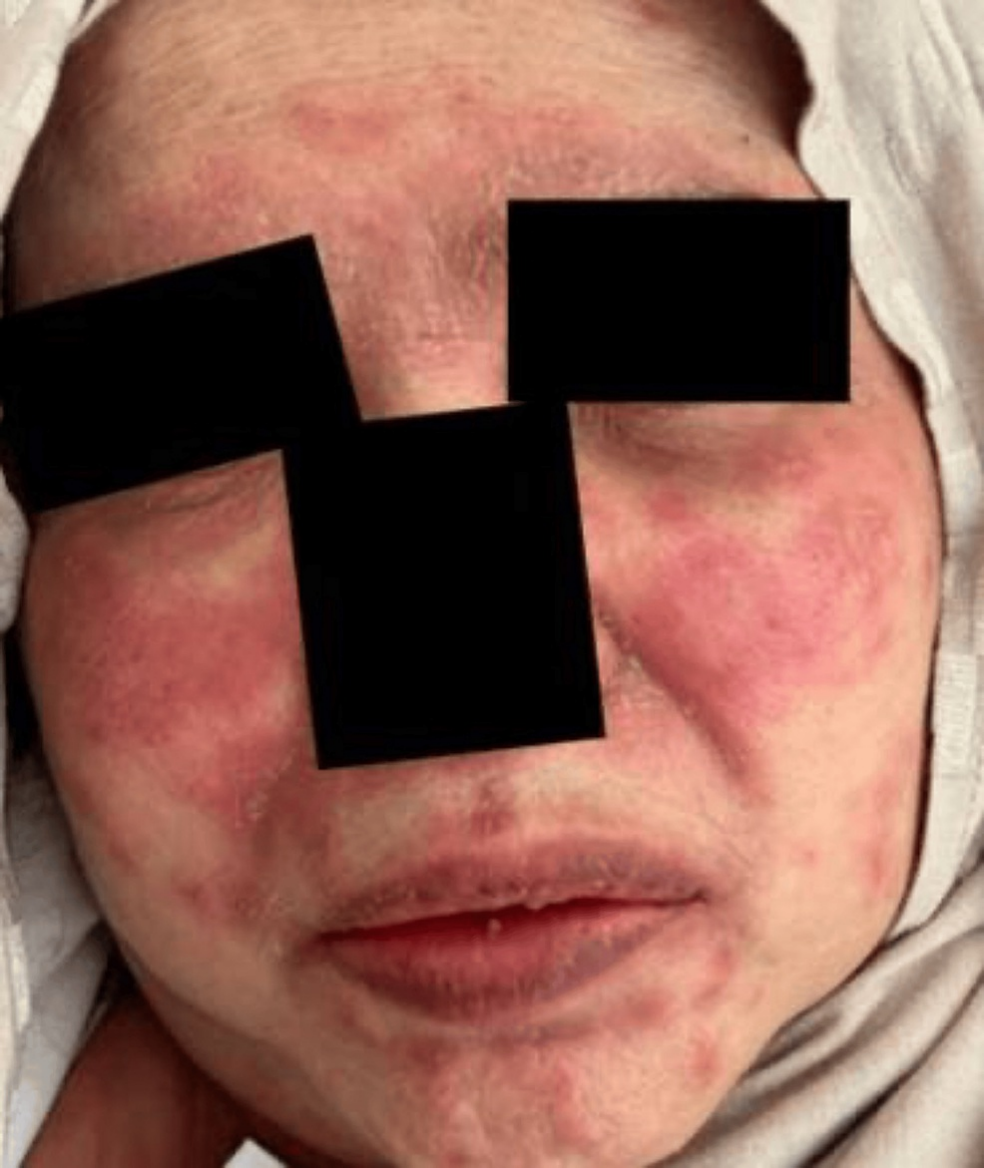
Urticarial wheals over the surfaces of the face, neck, ears, and trunk with lip swelling and crusts, as well as angioedema, present

**Figure 2:**
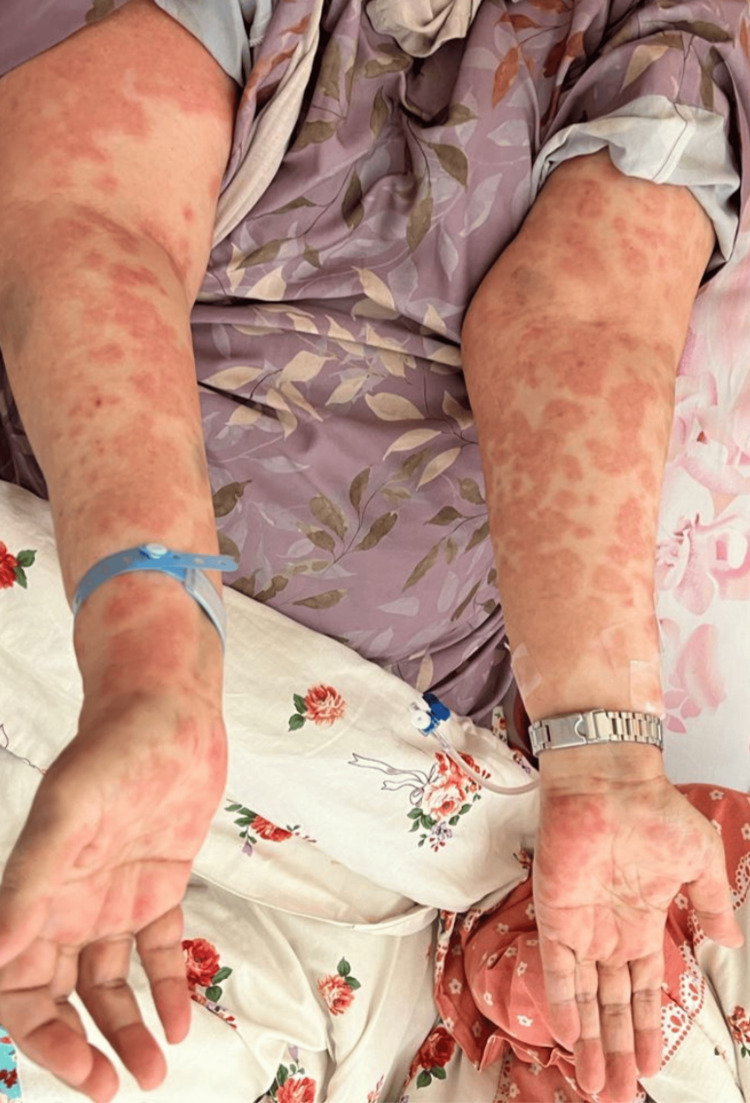
Non-scaly, erythematous, non-blanchable maculopapular rash over the upper extremities with palmoplantar involvement

**Figure 3:**
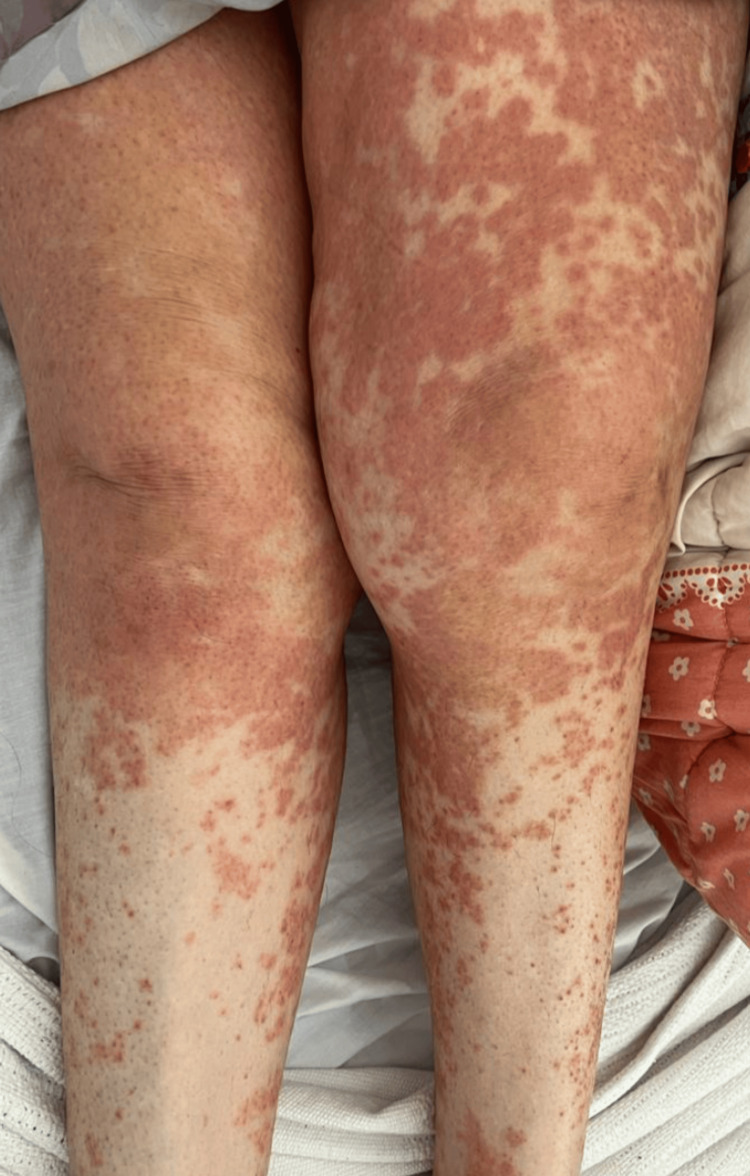
Generalised non-scaly, erythematous, non-blanchable maculopapular rash over the lower extremities with palmoplantar involvement

**Figure 4:**
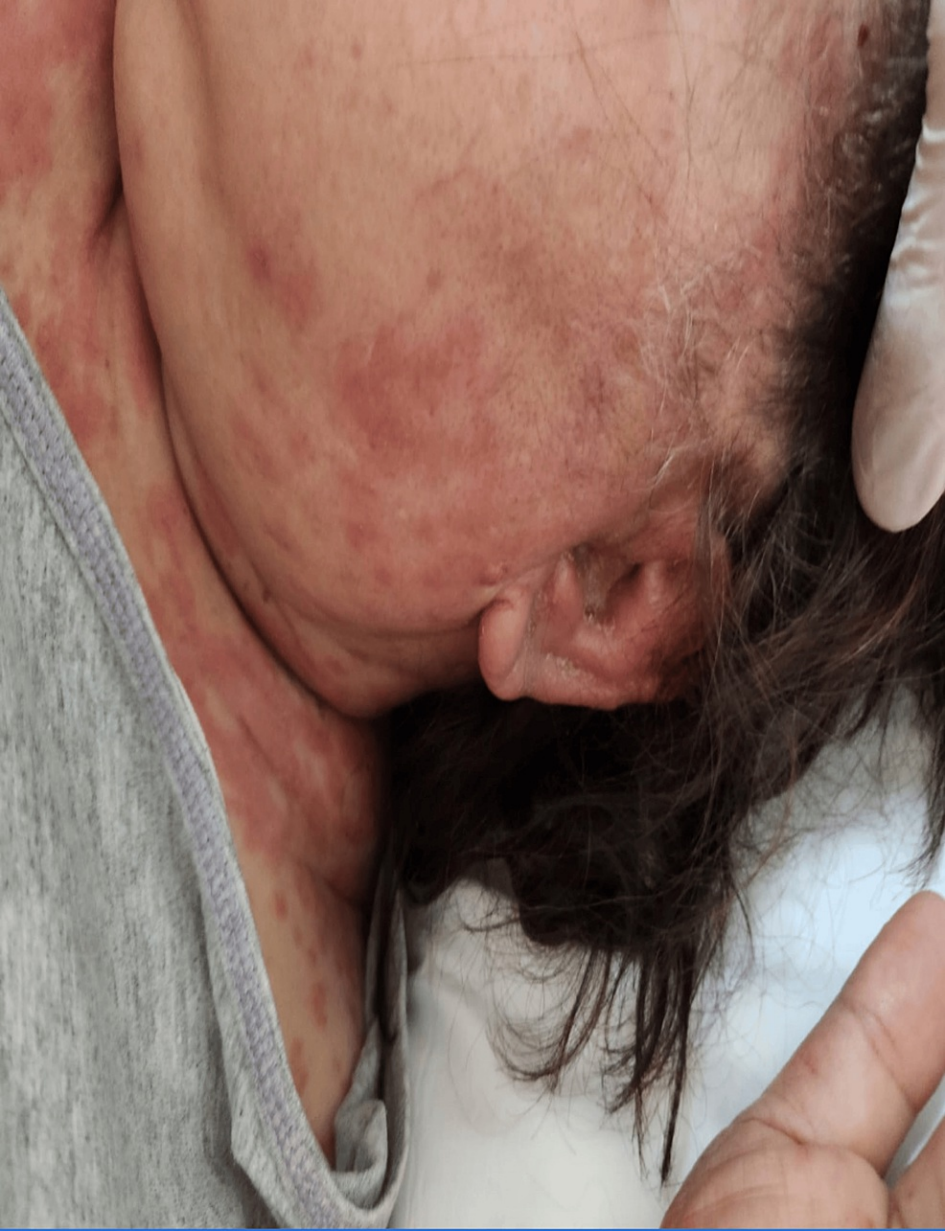
Bilateral otorrhea of watery substance

Upon examination, her blood pressure was 151/66 mmHg, oxygen saturation was 92% on room air, for which she was given 3 liters of oxygen through a nasal cannula, her temperature was 38.7 °C, heart rate was 177 beats per minute, respiratory rate was 20 breaths per minute, and her random blood glucose was 241 milligrams/deciliter. There were urticarial wheals over the surfaces of the face, neck, ears, and trunk (Figure 1) with lip swelling and crusts, as well as angioedema, present. Her lower limbs had a non-scaly erythematous maculopapular rash over the extremities that were non-blanchable and petechiae, and generalized erythematous papule, with palmoplantar involvement (Figures 2, 3). She had excessive otorrhea of watery substance bilaterally (Figure 4). There was no hair or nail involvement and no lymphadenopathy and the Nikolsky sign was negative as well.

Laboratory investigations showed the patient had a normal complete blood count panel. Hepatic and renal profiles were within normal levels as well. Anti-double stranded DNA (anti-DsDNA), anti-nuclear antibody (ANA), and complements C3 and C4 were negative.

During her hospital stay, she received intensive care and was managed by a multidisciplinary team. The patient received dexamethasone 8 milligrams intravenously, then hydrocortisone 50 milligrams intravenously every six hours for one week. After that, she was discharged with a 60 mg tapered dose of oral prednisolone and antihistamines. Moreover, the patient took a three-month course of doxycycline, 100 mg two times daily.

A differential diagnosis was established to be either urticaria, subacute lupus, vasculitis, drug reaction with eosinophilia and systemic symptoms (DRESS), or Steven Johnson's syndrome. Histopathology confirmed the diagnosis of severe angioedema and UV. It showed a normal epidermal layer, with underlying edema in the dermis, vascular endothelial swellings, and erythrocyte extravasation with perivascular inflammatory cell infiltrate (mainly lymphocytes and neutrophils). This is most likely secondary to adalimumab-adaz versus carbamazepine-induced hypersensitivity syndrome with urticarial vasculitis. 

It was recommended to avoid using aromatic anti-epileptic medications like carbamazepine, and especially to avoid using adalimumab-adaz along with other TNF blocker groups since it has been shown to cause severe allergic reactions and urticarial vasculitis, which can be fatal to the patient.

In addition to continuing regular follow-up visits at the dermatology, pulmonary, endocrine, orthopedic, and neurology clinics and, most importantly, maintaining patient clinical remission of HS by another biological agent is necessary for ensuring favorable outcomes.

## Discussion

Urticarial vasculitis (UV) is a rare type of leukocytoclastic vasculitis (LCV) that is also known as hypersensitivity vasculitis. It is an immune complex-mediated vasculitis that mostly affects the dermis's small blood vessels, manifested by palpable purpura and wheals primarily on the lower limbs. Extra-cutaneous symptoms, which include severe angioedema, cough, shortness of breath, sore throat, and dysphagia, occur rarely [4]. LCV may develop secondary to a side effect of medication such as NSAIDs, phenytoin, and anti-TNF-a, as well as infections, neoplasms, and systemic inflammatory disorders [5]. A definitive diagnosis is made with a skin biopsy, which typically shows perivascular and vascular leukocytic infiltrates in addition to fibrinoid necrosis [4]. Most LCV cases are idiopathic and self-limiting.

The mainstay of treatment is discontinuing the offending agent and controlling the underlying illness; rare occasions call for the use of high-dose steroids or immunosuppressive drugs [5]. In fact, our patient responded well to the TNF antagonist's discontinuation and the start of 60 mg tapered prednisone and doxycycline 100 milligrams BID for three months as an anti-inflammatory agent. It is crucial to note that there have been instances in which prolonged, severe immunosuppressive medication has proven preferred such as in situations involving several organs. 

Adalimumab is an anti-TNF agent frequently used to treat moderate to severe hidradenitis suppurativa, and its use has been linked to the development of serious side effects, which are drug-induced autoimmune disorders such as systemic lupus erythematosus, cutaneous vasculitis, and lupus-like syndrome. Vasculitis is the most common autoimmune disease that results from anti-TNF therapy, which manifests one to three weeks after drug administration [6]. The distinctions between these various drug-induced autoimmune disorders are quite challenging. However, there are distinctions in the clinical and histopathological aspects that aid in diagnosis. Since our patient had a skin rash accompanied by multiple organ involvement, lupus was on our differential diagnosis list. However, on histopathological skin biopsy, the classically described features for the diagnosis of LCV make lupus less likely to be the cause of such presentation. Additionally, our patient did not meet the American Society of Rheumatology's (ASR's) criteria for systemic lupus. While wheals and angioedema are uncommon in lupus, the classic butterfly rash that gets worse with sun exposure does not appear in LCV patients.

Since vasculitis is the most common autoimmune disease that results from anti-TNF therapy, it was assumed that the adalimumab biosimilar was the root of LCV in this patient. This assumption was reinforced by the histopathology of the skin biopsy, which confirmed the diagnosis, and the disappearance of skin lesions after stopping anti-TNF.

Although it is known that the formation of antibodies against anti-TNF might result in immune-complex-mediated hypersensitivity vasculitis, the pathophysiology of LCV is unknown. It has been proposed that blocking the TNF-signaling pathway causes a cytokine imbalance and a change in the T helper 1 to T helper 2 cytokine pattern, which makes the patient more vulnerable to vasculitis [6].

Ramos-Casals et al. looked at 233 cases of autoimmune disorders induced by anti-TNFs [7]. Five of the 113 individuals who experienced vasculitis after receiving anti-TNF medication reported using adalimumab. The primary histopathologic finding in 52 of the patients was LCV. Data on adalimumab was sparse, however, there were 19 cases of LCV following etanercept and 11 cases after infliximab; purpura was the most common skin condition, followed by ulcerative lesions; and 90% of cases improved once the anti-TNF medication was stopped. Using alternative agents in the future may be an option, however, it is advisable to proceed cautiously when choosing an agent to replace the offending agent. Importantly, it should be emphasized that recent investigations have focused on the link between vasculitis and using ustekinumab [7].

To our knowledge, the current study is the first case describing vasculitis associated with anti-TNF therapy in Saudi Arabia, therefore, dermatologists should keep an eye out for this potential problem, histologic assessment, and thorough organ involvement studies must be performed if vasculitis linked to anti-TNF is suspected.

## Conclusions

Anti-TNF-alpha therapy remains a valuable treatment for hidradenitis suppurativa. One undesirable effect of adalimumab is urticarial vasculitis. In the majority of cases, full recovery takes place after stopping the offending substance, however, in some circumstances, corticosteroids and immunosuppressive drugs are necessary. Dermatologists need to be aware of the potential complications of adalimumab biosimilar to manage it effectively.
